# Toxic Responses of Different Shellfish Species after Exposure to *Prorocentrum lima*, a DSP Toxins Producing Dinoflagellate

**DOI:** 10.3390/toxins14070461

**Published:** 2022-07-05

**Authors:** Mei-Hua Ye, Da-Wei Li, Qiu-Die Cai, Yu-Hu Jiao, Yang Liu, Hong-Ye Li, Wei-Dong Yang

**Affiliations:** Key Laboratory of Aquatic Eutrophication and Control of Harmful Algal Blooms of Guangdong Higher Education Institute, College of Life Science and Technology, Jinan University, Guangzhou 510632, China; ymh18@stu2018.jnu.edu.cn (M.-H.Y.); daweili@jnu.edu.cn (D.-W.L.); caiqd2020@stu2020.jnu.edu.cn (Q.-D.C.); jiaoyh0809@jnu.edu.cn (Y.-H.J.); ly2019@stu2019.jnu.edu.cn (Y.L.); thyli@jnu.edu.cn (H.-Y.L.)

**Keywords:** DSP toxins, *Prorocentrum lima*, *Crassostrea gigas*, *Mytilus coruscus*, *Tegillarca granosa*, Nrf2 signaling pathway, esterification

## Abstract

*Prorocentrum lima* is a global benthic dinoflagellate that produces diarrhetic shellfish poisoning (DSP) toxins, which can be ingested by filter-feeding bivalves, and eventually pose a great threat to human health through food chain. After being exposed to *P. lima*, different bivalves may accumulate various levels of DSP toxins and display different toxic responses. However, the underlying mechanism remains unclear. Here, we found that the content of okadaic acid-equivalents (OA-eq) varied in the digestive glands of the three bivalves including *Crassostrea gigas*, *Mytilus coruscus* and *Tegillarca granosa* after *P. lima* exposure. The degree of esterification of OA-eq in the three bivalves were opposite to the accumulation of OA-eq. The digestive gland tissues of the three bivalve species were damaged to different degrees. The transcriptional induction of Nrf2 targeted genes such as *ABCB1* and *GPx* indicates the functionality of Nrf2 pathway against DSP toxins in bivalves. The oyster could protect against DSP toxins mainly through ABC transporters and esterification, while the mussel and clam reduce the damage induced by DSP toxins mainly by regulating the expression of antioxidant genes. Our findings may provide some explanations for the difference in toxic response to DSP toxins in different shellfish.

## 1. Introduction

As filter-feeding mollusks, bivalves such as mussels, oysters, scallops and clams, can accumulate phycotoxins by filtering toxic algae, thus posing a great threat to human beings through the food chain [1]. Okadaic acid (OA) and its derivatives (dinophysistoxins (DTX)) are mainly responsible for diarrhetic shellfish poisoning (DSP) in human beings, which can be produced by some algal species in the genera *Prorocentrum* and *Dinophysis*, such as *Prorocentrum lima*, *P*. *concavum* and *P.* *maculosum* [2]. These toxins can cause human gastrointestinal diseases, such as diarrhea, vomiting and abdominal pain. Hence, they are known as DSP toxins. Many toxicological studies have reported the toxic effects of OA in cytotoxicity, genotoxicity, immunotoxicity, embryotoxicity, neurotoxicity and carcinogenicity [3].

The ability of bivalves to accumulate and remove DSP toxins varies among different species, which may be related to the feeding behavior, assimilation, excretion and biotransformation/detoxification of toxins [4,5]. In addition, other variables such as temperature and food absorbability also affect the detoxification rate of DSP toxins, as they directly affect the metabolic activity of bivalves [6]. Bivalve mollusks are able to biotransform accumulated toxins to produce some toxin metabolites that are not found in toxic algae [7]. The main conversion pathway is esterification with fatty acids of different chain lengths, which may facilitate the elimination of toxins [8]. Compared with other bivalves, the mussel *Mytilus galloprovincialis* was found to have a higher potential in OA accumulation, which was attributed to the mussel’s lower ability to eliminate OA [4]. Studies have shown that under the same circumstances, OA in oysters mainly exist in the form of esters (>90%), while the ester derivatives in mussels account for only a small part of the total amount of OA. Compared with mussels (21–41%), OA is more esterified in oysters (83–93%) [9]. However, the mechanism for the differences in the accumulation of DSP toxins in different shellfish is still largely unknown.

Many studies have shown that bivalve mollusks have developed a cytoprotective mechanism to reduce the harmful effects of algal toxins and that phase I metabolic enzymes (such as cytochrome P450 (CYP450), phase II metabolic enzymes (such as glutathione-S-transferase (GST), phase III transporter (such as P-glycoprotein (P-gp) and antioxidant system enzymes (superoxide dismutase (SOD); peroxidase (CAT)) may be involved in this process [2,10,11,12,13,14,15,16]. P-gp, a member of the ATP-binding cassette (ABC) family, mediates the multixenobiotic resistance mechanism (MXR) in aquatic organisms together with the multidrug resistance associated protein (ABCC/MRP) and the breast cancer resistance protein (ABCG2/BCRP). The MXR is a defense mechanism against environmental pollution that pumps various xenobiotics out of the cell and plays an important role in the metabolic detoxification of shellfish [17]. The Nrf2-Keap1 pathway also exists in bivalves [18] and plays an important role in protection against DSP toxins-induced oxidative damages by regulating the expression of targeted genes [16,19].

The bivalves *Crassostrea gigas*, *M. coruscus* and *Tegillarca granosa* are important economic shellfish in China. The DSP toxin accumulation was great difference in mussel, clam and oyster, which suggests that these three bivalves species may accumulate different levels of DSP toxins [20]. However, there are few studies that have dealt with the toxin’s accumulation and toxic response of the three species to *P. lima* and underlying mechanism remains largely unclear. Our objectives are to reveal the differences in DSP toxins accumulation in these three shellfish and explore the underlying mechanism. To this end, we observed the accumulation of DSP toxins, the level of esterification and morphological changes in the digestive glands of the three bivalves after *P. lima* exposure. Changes in the expression of some genes related to the Nrf2 signaling pathway were also analyzed.

## 2. Results

### 2.1. OA Content in the Digestive Gland Tissues of Different Bivalves Exposed to Prorocentrum lima

**Figure 1:**
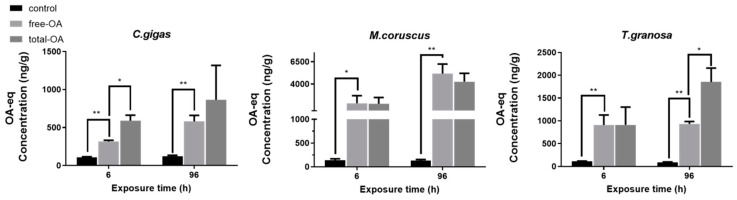
Content of free OA-eq and total OA-eq in the digestive gland of the three bivalves after exposure to *Prorocentrum lima.* *n*=3 replicates with 8 individuals per replicate. *t*-test, * *p* < 0.05, ** *p* < 0.01.

In this paper, we used *P. lima* (No. 2579) as source of DSP toxins and determination of LC-MS/MS showed that OA and DTX1 were produced (Figure S1). Considering the availability and ease of operation of an Okadaic Acid (DSP) ELISA Kit, we used this kit to determine the content of DSP toxin. Since this kit measures the ensemble of OA, DTX1 and DTX2, we use OA-equivalent (OA-eq) to represent the content of DSP toxins. As shown in Figure 1, the content of free OA-eq in the digestive gland of the oyster *Crassostrea gigas* was 320 ± 20 ng/g and 580 ± 80 ng/g at 6 h and 96 h, respectively, after exposure to *P. lima*, significantly higher (*p* < 0.01) than that of the control (110 ± 10 ng/g at 6 h and 120 ± 20 ng/g at 96 h). The total OA-eq content in the digestive gland of *C. gigas* was 590 ± 70 ng/g and 900 ± 500 ng/g, respectively, significantly higher than that of free OA-eq (*p* < 0.05) after exposure for 6 h, but not for 96 h.

The content of free OA-eq in the digestive gland of the mussel *Mytilus coruscus* was 1800 ± 900 ng/g and 5000 ± 1000 ng/g after 6 h and 96 h exposure of *P. lima,* significantly higher than that of the control (140 ± 30 ng/g and 130 ± 30 ng/g) (*p* < 0.01). The total OA-eq content (1800 ± 700 ng/g at 6 h and 4300 ± 900 ng/g at 96 h) was not significantly different from the free OA-eq content (*p* > 0.05).

By contrast, with *C. gigas* no significant difference was found at 6 h (*p* > 0.05) in the clams *Tegillarca granosa* between total OA-eq and free OA-eq after exposure to *P. lima*, while the total OA-eq (1900 ± 300 ng/g) in the digestive gland was significantly higher than the free OA-eq (940 ± 50 ng/g) at 96 h after exposure. The content of free OA-eq in the digestive gland of the clams *T. granosa* at 6 h and 96 h was 900 ± 200 ng/g and 940 ± 50 ng/g, respectively, significantly higher than that of the control (110 ± 10 ng/g at 6 h and 90 ± 10 at 96 h) (*p* < 0.01).

**Figure 2:**
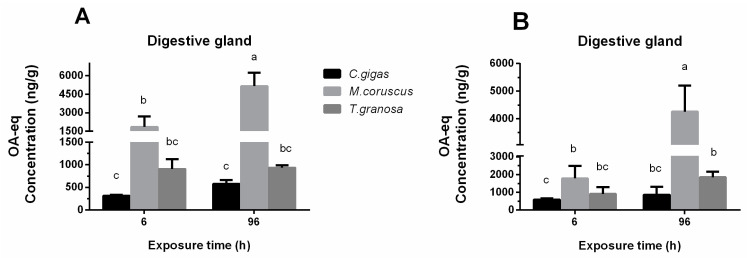
Comparison in contents of free OA-eq (**A**) and total OA-eq (**B**) in the digestive gland of the three bivalves after exposure to *Prorocentrum lima.* *n* = 3 replicates with 8 individuals per replicate. Bars of respective treatment followed by the same letter are not significantly different at *p* < 0.05 (Duncan’s test).

There were significant differences in the content of free OA-eq in digestive gland tissues of different shellfish after exposure to *P. lima*. At 6 h after exposure, the content of free OA-eq in digestive gland tissues of mussels was 1800 ± 900 ng/g, significantly higher than that of oysters (320 ± 20 ng/g) (*p* < 0.05), but there was no significant difference between mussels (1800 ± 900 ng/g) and clams (900 ± 200 ng/g). At 96 h, the content of free OA-eq in mussels (5000 ± 1000 ng/g) was the highest, significantly higher than that in clams (940 ± 50 ng/g) and oysters (580 ± 80 ng/g), but there was no significant difference in the content of free OA-eq in oysters and clams (Figure 2A). Meanwhile, at 6 h after exposure, the content of total OA-eq in digestive gland tissue of mussels was 1800 ± 700 ng/g, significantly higher than that of oysters (590 ± 70 ng/g) (*p* < 0.05). At 96 h, the total OA-eq content (4300 ± 900 ng/g) in mussel was distinct higher than those in clams (1900 ± 300 ng/g) and oysters (900 ± 500 ng/g) (*p* < 0.05).

### 2.2. Morphological Changes in the Digestive Glands of the Three Bivalves after Prorocentrum lima Exposure

After exposure to *P. lima*, the digestive glands of the three shellfish were damaged to varying degrees. Among them, the clam *Tegillarca granosa* suffered the most damage with about 20% intermediate alteration and 40% severe digestive tubules alterations, followed by the mussel *Mytilus coruscus* (about 60% intermediate damage) and the oyster *Crassostrea gigas* (about 20% light alteration) as described in Table 1.

**Figure 3:**
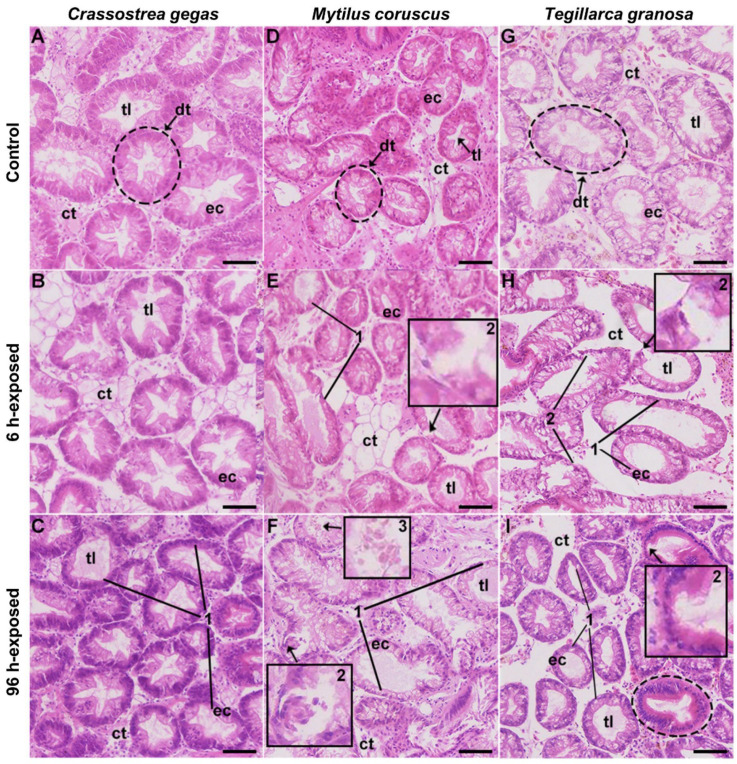
Digestive gland tissue section of *Crassostrea gigas*, *Mytilus coruscus* and *Tegillarca granosa* after exposure to *Prorocentrum lima* (HE staining). (**A**,**D**,**G**) Normal structure of the digestive gland of *C. gigas*, *M. coruscus* and *T. granosa* (fed with *Tetraselmis subcordiformis*). The digestive tubules (dt) were constituted by a single layer of orderly distributed ciliated eosinophilic epithelial cells (ec) with a narrow tubule lumen (tl). The connective tissues (ct) were comprised of few cells, such as fibrocytes, hemocytes and typically hyalinocytes. (**B**) No obvious damage was observed in *C. gigas* after exposure to *P. lima* for 6 h, with dilatation trend of individual tubule lumen (tl). (**C**) As shown by mark 1, atrophied epithelial cells were observed locally in *C. gigas* after exposure to *P. lima* for 96 h, in addition to increased volume of tubule lumen (tl). (**E**) After exposure to *P. lima* for 6 h, the tubule lumen (tl) dilated and the epithelial cells (ec) became thinner and even deformed in *M. coruscus*, as shown by mark 2. (**F**) After exposure to *P. lima* for 96 h, the tubule lumen (tl) dilated and the epithelial cells (ec) became thinner in *M. coruscus* and hemocytic infiltration were observed inside the tubule lumen (tl) as shown by mark 3. (**H**) Dilated tubule lumen (tl) and thinner epithelial cells (ec) were observed in *T. granosa* after exposure to *P. lima* for 6 h. In addition, there are more decomposed epithelial cells as shown by mark 2. (**I**) Atrophy of the epithelial cells led to the deformed digestive tubules (dt). Mark 1, thinner epithelial layer. Mark 2, decomposed epithelial cells. Mark 3, hemocytic infiltration. Scar bar, 50 μm.

The cross-sections of digestive glands in the three species of bivalves without *P. lima* exposure presented normal morphology, consisting of digestive tubules (digestive diverticula) formed by a single layer of ciliated epithelial cells, with an almost occluded lumen (Figure 3A,D,G). In addition, the columnar epithelial cells were neatly arranged without atrophy and the connective tissue was formed by uniformly distributed fibrocytes and hemocytes. In general, no obvious damage was observed. After exposure to *P. lima*, the digestive glands of the three shellfish were damaged to varying degrees, mainly manifested as dilated tubule lumen, atrophied epithelial cell, deformed digestive diverticulum and hemocytic infiltration, as described in previous study [21].

In the *P. lima*-exposed *C. gigas*, the damage was minor and the tubule lumen was slightly dilated at 6 h (Figure 3B), while in individual samples atrophied epithelial cells were observed locally at 96 h (Figure 3C). The dilated tubule lumen was more common, which might be attributed to more serious epithelial atrophy in *M. coruscus* after exposure to *P. lima* for 6 h (Figure 3E). The height of epithelial cells was significantly reduced which formed the thinner epithelial layer. Meanwhile, some digestive diverticulum appeared deformation. The tubule lumen dilation and epithelial cells thinning became more severe in *M. coruscus* after exposure to *P. lima* for 96 h and hemocytic infiltration were observed inside the tubule lumen (Figure 3F). In addition to the main damages observed above, decomposed epithelial cells were universal distributed in *T. granosa* after exposure to *P. lima* for 6 h (Figure 3H). At 96 h, the continuous atrophy of epithelial cells led to the deformed digestive tubules (Figure 3I).

### 2.3. Expression Changes of Nrf2 Signal Pathway and Related Genes in Digestive Gland Tissues of Different Shellfish Exposed to Prorocentrum lima

**Figure 4:**
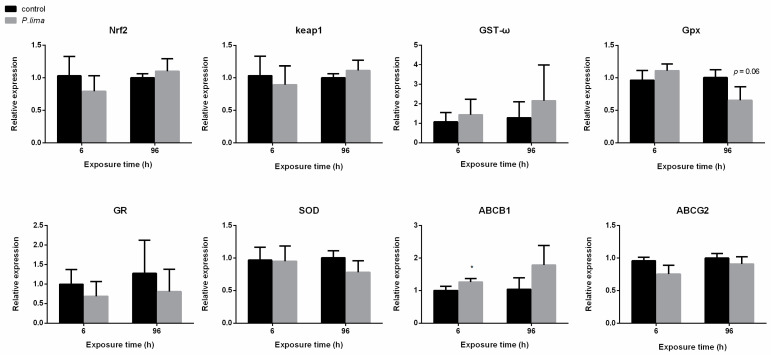
Changes in expression of Nrf2 signaling pathway-related gene in the digestive gland of *Crassostrea gigas* after exposure to *Prorocentrum lima* revealed by qPCR. *n* = 3 replicates with 8 individuals per replicate. *t*-test, * *p* < 0.05.

**Figure 5:**
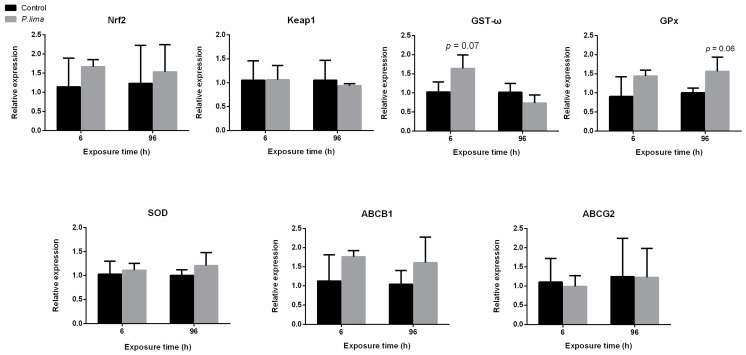
Changes in expression of Nrf2 signaling pathway-related gene in the digestive gland of *Mytilus coruscus* after exposure to *Prorocentrum lima* revealed by qPCR. *n* = 3 replicates with 8 individuals per replicate. *t*-test.

**Figure 6:**
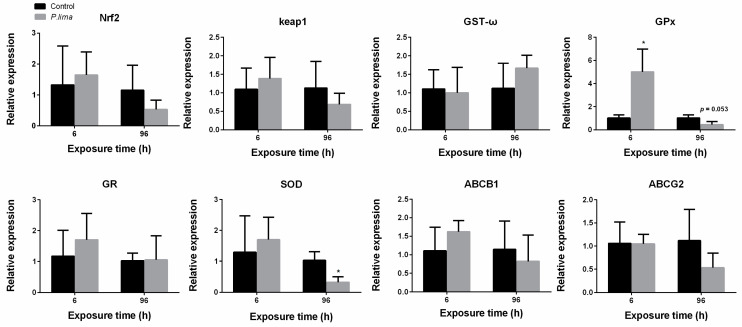
Changes in expression of Nrf2 signaling pathway-related gene in the digestive gland of *Tegillarca granosa* after exposure to *Prorocentrum lima* revealed by qPCR. *n* = 3 replicates with 8 individuals per replicate. *t*-test, * *p* < 0.05..

As shown in Figure 4, the expression of *ABCB1* in the digestive gland of the oyster was significantly up-regulated (*p* < 0.05) at 6 h after exposure to *P. lima*. There was no significant change in the expression of *Nrf2, ABCG2, SOD, GST-ω*, glutathione peroxidase (*GPx*) and *GR*. After exposure to *P. lima*, the expression of *GST-ω* at 6 h and *GPx* at 96 h did not change significantly (Figure 5), rather it showed an up-regulation trend in the digestive gland tissues of the mussel (*p* > 0.05). There was no significant change in the expression of *Nrf2*, *ABCB1*, *ABCG2* and *SOD*. In the case of clams, *GPx* transcription was significantly up-regulated at 6 h (*p* < 0.05), while *SOD* was significantly down-regulated at 96 h after exposure of *P. lima*. The expression of other genes did not change significantly after exposure to *P. lima* (Figure 6).

## 3. Discussion

The differences in DSP toxin accumulation in different shellfish are due to many factors, including ingestion behavior, pseudo-feces production and esterification. However, the esterification ability in different bivalves is different. In our experiments, we found that the OA-eq level in the digestive glands varied in different shellfish after exposure of *Prorocentrum lima*. The digestive glands of *Mytilus coruscus* had the highest level of OA, followed by *Tegillarca granosa* and *Crassostrea gigas* accumulated the least toxins. The proportion of esterified toxins in *C. gigas* and *T. granosa* was higher, while that in mussels was very low. Compared to *T. granosa*, *C. gigas* had a faster esterification rate. Similar to our results, studies have shown that the European flat oyster (*Ostrea edulis*) contained a high proportion of esterified OA (86%) [22], while only 41% of the OA in *Mytilus edulis* was esterified [9]. The esterification rate of OA and DTX2 of *Cardium edule* was higher than that of *Mytilus edulis* [23]. In our experiment, three kinds of shellfish were fed the same food and cultured under the same conditions. The significant differences in DSP toxins of the three kinds of shellfish were mainly caused by the different esterification ability and metabolism of DSP toxins and pseudo-feces production may also play some roles. Unfortunately, we could not observe the pseudo-feces formation of the three shellfish after exposure to *P. lima*. In our experiment, animals in the *P. lima*-exposed group were fed 2 × 10^6^ cells/L of *P. lima* and 1 × 10^7^ cells/L of *Tegillarca* *subcordiformis*, in which pseudo-feces and feces were mixed together. Therefore, it is difficult to assess pseudo-feces production.

The Nrf2/ARE signaling pathway is an important pathway for the body to protect from oxidative damage induced by external compounds. Several Nrf2/ARE signaling pathway target genes such as *GST*, ABC transporters, *SOD* and catalase *(CAT)* have been suggested to be involved in the metabolic detoxification of DSP toxins in bivalves [19]. We found that the expression of *ABCB1* in the digestive gland of the *C. gigas* and *GPx* in the digestive gland of the *T. granosa* were significantly upregulated at 6 h. However, no significant changes were observed in the expression of *Nrf2* and *Keap1* in the three mussels after exposure to *P. lima*. Similar to our results, a study reported that exposure to curcumin (30 μM, 24 h) resulted in a significant increase in antioxidant capacity in gill tissue of *C. gigas*, while mRNA expression levels of Nrf2 signaling pathway-related genes *GR*, *GPx2* and *GSTpi* were significantly upregulated, but neither Nrf2 nor Keap1 genes were significantly changed [18].

The antioxidant enzyme system plays an important role in the defense against oxidative stress, which includes SOD, CAT, GST and GPx, etc. It has been suggested that CYP450, GST and ABC transporter proteins may be involved in the biotransformation and detoxification of DSP toxins [24]. One study showed significant changes in *SOD*, *CAT* and *GST-pi* expression in the digestive gland of *Mytilus galloprovincialis* after exposure to *P. lima* [25]. In our study, up-regulation of *GST-ω* in the digestive gland tissue of *M. coruscus* at 6 h and *GPx* at 96 h and *GPx* in *T. granosa* at 6 h after exposure to *P. lima*, suggest that the mussel *M. coruscus* and the clam *T. granosa* mainly defense against DSP toxins by regulating the expression of antioxidant genes, even Nrf2 was not changed after *P. lima* exposure.

P-glycoprotein is believed to play an important role in bivalves’ tolerance to exogenous chemicals [26]. Keppler proposed that OA was a substrate of *ABCB1*, so the accumulation of DSP toxins could induce the expression of *ABCB1* [27]. After 6 h of *P. lima* exposure, up-regulation of *ABCB1* in the digestive gland of *C. gigas* suggested that ABC transporter contributed to the detoxification in *C. gigas*. When *C. gigas* were exposed to *P. lima* for 96 h, there was no significant change in *ABCB1* expression, possibly because the protein encoded by *ABCB1* was sufficient to respond DSP toxins. Esterification is an important step in the depuration of DSP toxins [28]. The esterification of DSP toxins can increase the liposolubility of the toxins. Fat-soluble toxins, as substrates for ABCB1 protein transport, are more easily excreted from the body [28]. The esterification of DSP toxins and the enhanced expression of ABC transporter in *C. gigas* makes the toxins easily excreted and, therefore, *C. gigas* accumulated the lowest level of DSP toxins compared to *M. coruscus* and *T. granosa*.

Bivalves have evolved many pathways to reduce the effects of DSP toxins, including transformation (like esterification), MXR and antioxidant systems, etc. [5]. Even so, shellfish can be damaged to some extent after DSP toxins exposure. The changes in histomorphology can directly reflect the physiological alteration of shellfish exposed toxic substances [29]. Neves et al. have described the histological changes induced by DSP toxins in detail, featured in the tubular atrophy of digestive gland diverticula, the hemocyte infiltration, the reduction of digestive cells and the atrophy and decomposition of epithelial cells [30]. Similar to these findings, digestive glands displayed varying degrees of damage in the three bivalve species after exposure to *P. lima*, which might be due to the difference in toxin accumulation. The oyster *C. gigas* accumulated the lowest level of DSP toxins compared to *M. coruscus* and *T. granosa*, correspondingly, it suffered minor damage, suggesting that esterification of DSP toxins may be beneficial to the toxin elimination from bivalves. However, the damage of digestive gland in *T. granosa* was the most serious, but the toxin content was not higher than that in *M. coruscus*. This indicates that metabolic detoxification of DSP toxins may vary greatly among different bivalve species except for esterification. Unfortunately, we only measured the content of OA-eq and did not analyze accurately the components of DSP toxins due to the limitation of detect assay. In fact, *P. lima* can produce other toxins apart from DSP toxins [31]. In addition, DSP toxins can be metabolized in shellfish with different degree [5]. All these complicates the toxic response of different bivalve species to *P. lima*.

## 4. Conclusions

In summary, there were great differences in DSP toxins accumulation and toxic response between the three shellfish species after exposure to *Prorocentrum lima*. The oyster *Crassostrea gigas* could resist DSP toxins mainly through ABC transporter proteins and esterification, while the mussel *Mytilus coruscus* and the clam *Tegillarca granosa* reduced the damage induced by DSP toxins mainly by regulating the expression of antioxidant genes. The transcriptional induction of Nrf2 targeted genes such as *ABCB1* and *GPx* indicates the functionality of Nrf2 pathway against DSP toxins in bivalves, despite the lack of evidence of a transcriptional regulation of Nrf2. However, the toxicity of shellfish to *P. lima* was closely related to algal density and varied with exposure time. In this paper, we only analyzed the expression of the Nrf2 signaling pathway under one algal density and two time points. Therefore, further observation of the relationship between algal density and effect and the relationship between time and effect are necessary to reveal the causes of the difference in response of different shellfish to toxins.

## 5. Materials and Methods

### 5.1. Acclimatization and Algal Production Phases

The experimental animals *Mytilus coruscus* (total length: 9 ± 1 cm), *Crassostrea gigas* (total length: 13 ± 2 cm) and *Tegillarca granosa* (total length: 3.30 ± 0.80 cm) were purchased from the Huangsha Aquatic Products Market in Guangzhou, China. Individuals with moderate sizes were selected and transported to the laboratory quickly as soon as possible under low temperature. After being transported to the laboratory, the animals were cleaned with artificial seawater to remove the parasites attached to the shells and excess mud, then acclimated in a series of tanks with 5 L of clean sea water (with a salinity of 32.0 ± 1) per tank. The size of the animals was determined with calipers. The animals were cultured in a temperature-controlled incubator with 18 ± 1 °C under a fixed photon flux density and a 12 h light: 12 h dark cycle and an oxygen pump was equipped to continuously supply oxygen. The animals were fed with 1 × 10^7^ cells/L of *Tetraselmis subcordiformis*. Culture water was renewed once a day at set time.

The *Prorocentrum lima* (No. 2579) used in this experiment was kindly provided by National Center for Marine algae and Microbiota (NCMA), which has been proved to produce DSP toxins [32,33]. The chlorophyte *T. subcordiformis* was purchased from Institute of Hydrobiology, Chinese Academy of Sciences. The two algal species were cultivated in batch cultures at 20 ± 1 °C with a modified silicon-free f/2 medium under a cool-white fluorescent illumination of 40 μmol/(m^2^ s) with a 12 h/12 h photoperiod.

### 5.2. Experimental Design

After 7 days of acclimatation, individuals with good physiological conditions (can respond quickly and close their shells tightly when stimulated), strong vigor and uniform size were randomly selected for experiment. A total of 96 individuals of each type of shellfish were divided into two groups. Cultural water was regularly replaced once a day and new microalgae were added as did during acclimatation. Animals in the *P. lima*-exposed group were fed with 2 × 10^6^ cells/L *P. lima* and 1 × 10^7^ cells/L *T. subcordiformis* in each tank, respectively, and those in the control group were fed only with 1 × 10^7^ cells/L *T. subcordiformis* in the tank. After *P. lima* exposure for 6 h and 96 h, digestive gland tissues were taken from 24 individuals in each group at each time point and every 8 animals were pooled together as one sample to form 3 samples (replicates). The individual from each tank were sacrificed and dissected. Each sample was divided into 3 parts, which were used for RNA extraction, toxin determination and tissue section. The part used for RNA extraction was quick-frozen with liquid nitrogen and stored in the refrigerator at −80 °C, the sample used to determine the concentration of DSP toxins was stored in the refrigerator at −20 °C and that for tissue section was placed in Bonn fixative.

### 5.3. Determination of the Total OA-eq Content and Free OA-eq Content 

Toxins’ extraction and detection in samples were performed as described in our previous paper [13]. An Okadaic Acid (DSP) ELISA kit (Abraxis, Warminster, PA, USA) was used to detect the total OA-eq content and free OA-eq content in digestive gland tissues [34]. The degree of cross-reactivity with the DSP toxins in this kit was as follows: OA (100%), DTX1 (50%) and DTX2 (50%). The absorbance at 450 nm was detected by a Synergy H1 Hybrid Multi-Mode Microplate Reader (Bio-Tek, Winooski, VT, USA) and the OA-eq content was calculated according to the standard curve generated. 

For the total OA-eq content, hydrolysis of samples was performed [35]. In brief, 100 μL NaOH (1.25 M) was added to 500 μL of toxin extract, and the resulting solution was incubated at 76 °C for 40 min. After cooling, the samples were neutralized with 100 μL HCl (1.25M), and the content of OA-eq was determined with the kit. The difference between total OA-eq content and free OA-eq content represents the content of esterified toxin. The content of DSP toxins was expressed as ng OA eq/g. 

### 5.4. qPCR

Total RNA was extracted from about 50 mg of samples by using a Total RNA Kit I (50) R6934-01 (Omega, Norcross, GA, USA) as described in our previous paper [36]. PCR was carried out on a CFX 96 Fluorescent PCR instrument (BioRad, Hercules, CA, USA) with AceQ^®^ qPCR SYBR^®^ Green Master Mix (Q111-02, Vazyme, Nanjing, China). PCR profile was as follows: 95 °C for 5 min, 40 cycles of 95 °C for 10 s, 60 °C for 30 s. PCR specificity was evaluated by melting curve analysis from 65 °C to 95 °C, increasing by 0.5 °C at 5 s. Reaction mixture (20 µL) consisted of 10 µL of AceQ^®^ qPCR SYBR^®^ Green Master Mix, 8.2 µL of H_2_O, 0.4 µL of each forward and reverse primers (10 µM) and 1 µL of first-strand cDNA template from 1 µg RNA. During the experiment, three replicate wells were set to ensure the reproducibility of PCR (technical replicates) and no-reverse transcriptase (NRT) was set for error correction between different plates. Amplification was also performed on equivalent double-distilled water as no template control (NTC) to check the absence of contaminant. The amplification efficiency of all genes was between 90% and 106.8% and the correlation coefficient was above 0.988. 

Three software programs, geNorm (version 3.4), Normfinder (v 0.953) and bestkeeper (version 1) were used to screen the internal reference genes. In the *C. gigas*, glyceraldehyde-3-phosphate dehydrogenase (*GAPDH*) and α-tubulin were screened from the five genes including *GAPDH*, elongation factor 1 alpha (*EF1α*), α-tubulin, ribosomal protein L 17 (*rpl17*) and *18S*. In the *M. coruscus*, α-tubulin and *18S* were screened as internal reference genes from *GAPDH*, ribosomal protein S23 (*rps23*), *18S*, α-tubulin and *EF1α*. In the *T. granosa*, *GAPDH* and α-tubulin were screened from the four genes *GAPDH, EF1α*, α-tubulin and *18S*. All the primers were designed based on the sequences of three bivalves by using Primer 5.0 (Table 2, Table 3 and Table 4).

The comparative C_q_ method was used to analyze the relative expression level of genes by using multi-internal reference genes method [37].

### 5.5. Histological Examination

Histological examination was performed as in previous studies with some modifications [38]. Briefly, digestive gland tissue was carefully dissected from animals and fixed in 10 mL of Boone’s fixative for at least 48 h at room temperature. After paraffin embedding, the tissue was sectioned at 4 µm thickness using an RM2235 paraffin microtome (Leica, Heidelberger, Nuβloch, Germany). The section obtained were then stained with hematoxylin–eosin and imaged using the Digital Section Scanning and Application System (Motic, Xiamen, China). The picture was analyzed by Motic VM 3.0 (Motic, Xiamen, China). According to previous studies [30,39], histological changes of digestive glands was evaluated and the damage was classified into normal, slight, intermediate and severe degrees.

### 5.6. Statistical Analyses

All data were analyzed by using IBM SPSS Statistics 22.0 statistical software and given as mean ± standard deviation (SD). Independent samples t-test was used and the homogeneity of variance was tested. In addition, Duncan’s test was performed for comparison of the free OA-eq and total OA-eq (B) levels between different bivalves with a significant difference at *p* < 0.05.

## Figures and Tables

**Table 1:** Pathological conditions in digestive tubules of different bivalves after exposure to the toxic dinoflagellate *P. lima* (*n* = 5).

Species	Exposure Time (h)	Light	Moderate	Severe
*Crassostrea gigas*	6	+(1/5)	−(0/5)	−(0/5)
	96	+(1/5)	−(0/5)	−(0/5)
*Mytilus coruscus*	6	+(1/5)	+(2/5)	−(0/5)
	96	+(2/5)	+(4/5)	−(0/5)
*Tegillarca granosa*	6	+(1/5)	+(1/5)	+(1/5)
	96	+(2/5)	+(1/5)	+(3/5)

1/5 means that one of the five animals was injured and so on.

**Table 2:** The primers used for the qPCR of *Tegillarca granosa*.

Gene Name	Primer Sequence	Fragment Size
*Nrf2*	F: AGAGCAACAGCGACAACAGGAAC	145
	R: AGCTTGTGGTGGCATTTGAGGAG	
*Keap1*	F: ACGGAATCGAGTGGGAGTTGGAG	80
	R: AGTTATGGTGAGTCTGCCCCTGAG	
*SOD*	F: GGCCAGCATGGGTTCCATATCC	109
	R: CGTCTTCTGGTCCACCATGTTCC	
*GR*	F: GGTCGGGAGGTTTGGCAAGTG	89
	R: ACATGTGCCACCCCATTTCCC	
*ABCG2*	F: CTGGGACCAACAGGAAGTGGAAAG	149
	R: TCATCCTGAACCACATAGCCAACC	
*ABCB1*	F: GATGGCTTCTTTTGGGCAATCTGG	142
	R: TGTTGCCAAACGGGTAGTCATAGC	
*GPx*	F: CTACGAGAACGACTGCCGACAC	150
	R: TCTTTTGTATGGCTTCCCGTCTGG	
*GST-ω*	F: AAATCGTTAGGTGAGAGGGGAG	101
	R: TCTCCACGCATTCAGTTTCG	
*Tubulin*	F: ACCACTGCCATTGCTGAAGCC	108
	R: TCCCTCCTCCATACCCTCTCCTAC	
*18S*	F: CTTTCAAATGTCTGCCCTATCAACT	91
	R: TCCCGTATTGTTATTTTTCGTCACT	
*GAPDH*	F: GTTGGCAAGGTCATTCCAGCTTTG	135
	R: AGCTGCCTTGATTGCGTCATAGC	
*EF1α*	F: TGATGCCCCAGGACACAGAGAC	88
	R: ACCAGCAGCAACAATCAGGACAG	

**Table 3:** The primers used for the qPCR of *Crassostrea gigas*.

Gene Name	Primer Sequence	Fragment Size
*Nrf2*	F: CATCTGAGTGTGCTGGAGAACGAC	89
	R: ATGGGCTGATGGGAAGGTGAGG	
*Keap1*	F: CGGCTATGATGGCAGCAACAGG	124
	R: GCCTTCCATTCCGATCACACCA	
*SOD*	F: CTGGACGGCACTTTAACCCCTTC	92
	R: GCCAGCGGTGACATTACCAAGG	
*GR*	F: TCAGCCAAGCGGTTACAGACAATC	106
	R: TCCAGTGTTAGGGTGCCGTCTC	
*ABCG2*	F: CCCTCCGCCTTCCATCAAAACTG	143
	R: TTTACGCTCTCCTCCAGACACTCC	
*ABCB1*	F: GCGTCATCATCGGCTTCGTCTAC	110
	R: AACTCCCTCCAACAACTGCATCTG	
*GST-ω*	F: TGAGTTCACCACCGCAAGAGAAAC	140
	R: CCACAGCAGGAAGTCTAGCATCTG	
*GPx*	F: TGTCATCAACCAGCAGGGTAAACC	102
	R: CCACGGCAGAGTGAGGCAATAATG	
*EF1α*	F: TGCCACACTGCTCACATTGCC	150
	R: AACACACATAGGCTTGCTGGGAAC	
*RPL7*	F: GCTTCCGAGAGTGCCTGAAACC	91
	R: TTTGTTTTCGAGCTTTGCCTTGGC	
*18S*	F: ACACTGGACAACAAACTCCGTGAG	120
	R: TCTTCCTGTGGTCTTTGTGTGCTG	
*GAPDH*	F: AGGATTGGCGTGGTGGTAGAGG	82
	R: ATGACCTTTCCGACAGCTTTGGC	
*α-tubulin*	F: CCGCCAACTCTTCCATCCAGAAC	105
	R: ACCAAGTCCACGATCTCCTTCCC	

**Table 4:** The primers used for the qPCR of *Mytilus coruscus*.

Gene Name	Primer Sequence	Fragment Size
*Nrf2*	F: AGAGCAACAGCGACAACAGGAAC	105
	R: AGCTTGTGGTGGCATTTGAGGAG	
*SOD*	F: GGCCAGCATGGGTTCCATATCC	93
	R: CGTCTTCTGGTCCACCATGTTCC	
*ABCB1*	F: GATGGCTTCTTTTGGGCAATCTGG	102
	R: TGTTGCCAAACGGGTAGTCATAGC	
*GPx*	F: CGTTCCTAAGTTCCAGATGTTTTG	116
	R: AACATCTTCGTTCTTCAGTGGTG	
*GST-ω*	F: TTGGGAGATGGAAAGTTGCG	89
	R: TGCCCGTCTGTAAGGGTCTG	
*Keap1*	F: TGAATGTTATGAGCCCGACAAGGATG	80
	R: CTCCTACACCAACTCCACCTTCATG	
*ABCG2*	F: GCTGTTGCTAACTTGTGTATTGCTCTC	113
	R: CTTGCCCATTTCAGCCATTGTAACC	
*18S*	F: GACCTCGGTTCTATTTTG	88
	R: GGTATCTGATCGTCTTCG	
*rps23*	F: TCACAGCCTTCGTCCCTAA	102
	R: CCTTTGAACAGAGCCCAGA	
*EF1α*	F: CACCACGAGTCTCTCCCTGA	110
	R: GCTGTCACCACAGACCATTCC	
*α-tubulin*	F: TTGCAACCATCAAGACCAAG	105
	R: TGCAGACGGCTCTCTGT	
*GAPDH*	F: CCGCCATTAAAGCAGCCTCTGAG	98
	R: ACTCCTGTTGTCTCCACGGA AATC	

## Data Availability

The data in this study are available from the corresponding author upon request.

## Supplementary Materials

The following supporting information can be downloaded at: https://www.mdpi.com/article/10.3390/toxins14070461/s1, Figure S1: Toxins profile of *Prorocentrum lima*.
