# Supplementary material for: Toxic Responses of Different Shellfish Species after Exposure to Prorocentrum lima, a DSP Toxins Producing Dinoflagellate
Source: Toxins (Basel). 2022 Jul 5;14(7):461. doi: 10.3390/toxins14070461 (PMC9317551; doi:10.3390/toxins14070461)
Supplement: Supplementary file 1 [file toxins-14-00461-s001.zip › toxins-1797350-supplementary.pdf]

toxins

MDPI

Article

Toxic Responses of Different Shellfish Species after Exposure to *Prorocentrum lima*, a DSP Toxins Producing Dinoflagellate

Mei-Hua Ye, Da-Wei Li, Qiu-Die Cai, Yu-Hu Jiao, Yang Liu, Hong-Ye Li and Wei-Dong Yang *

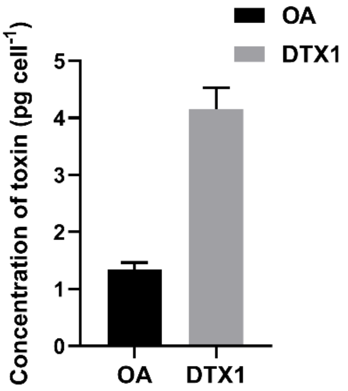

Figure S1. Toxins profile of *Prorocentrum lima.*
